# High-Resolution Real-Time Impulse Radar Using Software-Defined Radio: A Step-by-Step Implementation with USRP X310

**DOI:** 10.3390/s26175455

**Published:** 2026-08-28

**Authors:** Shekoufeh Abdollahi, Richard Bean, Neel Pandeya, Jihwan Yoon

**Affiliations:** 1 Electrical and Biomedical Engineering, University of Nevada Reno, Reno, NV 89557, USA; sabdollahi@unr.edu; 2 Bean Space Foundation, Reno, NV 89511, USA; rick@beanspacefoundation.org; 3 National Instruments (NI) Emerson Test and Measurement Group, Austin, TX 78759, USA; neel.pandeya@emerson.com

**Keywords:** software-defined radio, USRP X310, impulse radar, C++, radar

## Abstract

Software-defined radio (SDR) has become an attractive platform for radar research due to its flexibility, compact size, and ability to reconfigure radar methods for different situations. However, achieving high-resolution time-domain radar with SDR remains challenging due to demanding requirements for data throughput, sampling rate, and hardware configuration. Frequency-modulated radar implementations face similar limitations, often suffering from slow data acquisition and increased system complexity including exact waveform linearity, phase coherence, frequency-stepping control, and computationally intensive signal processing. In this work, a high-resolution impulse radar is implemented on a USRP X310 equipped with a UBX-160 daughterboard. The system fully exploits the hardware’s wide instantaneous bandwidth, enabling high range resolution in real time. Detailed hardware requirements and software configurations are presented step-by-step to ensure reliable operation at high data rates. To maximize performance and flexibility, the radar system is implemented using the low-level USRP Hardware Driver C++ API rather than graphical programming frameworks. This enables improved memory handling, reduced processing overhead, and precise timing control. The resulting modular design permits adaptability for different radar modalities with minimal code modification. The proposed platform provides a practical and reproducible framework for developing wideband, real-time SDR-based radar systems.

## 1. Introduction

Radio Detection and Ranging (radar) is a remote sensing technology that transmits electromagnetic waves and analyzes their reflections from objects. Radar determines the position, motion, and physical characteristics of targets in conditions where optical sensing is ineffective, such as poor lighting, long ranges, or propagation through opaque media. This ability makes radar an essential technology across a wide range of applications, including air traffic control, weather monitoring, defense surveillance, automotive safety, medical imaging, subsurface exploration, and military systems [1]. Over time, radar systems have evolved from large, hardware-intensive instruments to compact, specialized platforms, enabling opportunities for new research and commercial uses.

### 1.1. Limitations of Conventional Radar Systems

Conventional radar systems are typically built around fixed hardware architectures that are difficult to reconfigure. While these dedicated systems provide substantial benefits such as optimized RF chains and superior performance for their specific purpose, they suffer from inherent inflexibility [2].

Consequently, a radar system optimized for one application, such as long-range detection, may perform poorly in others, such as short-range high-resolution imaging. In many scenarios, switching between multiple radar methods is valuable, as each modality has particular strengths and limitations. For example, pulsed radar is effective for long-range detection with high peak power, while frequency-modulated continuous-wave radar (FMCW) provides better range resolution with lower peak power requirements. Switching between these methods for the same targets may optimize performance under varying conditions.

### 1.2. Software-Defined Radio as a Radar Platform

The need for flexible, multi-modal radar hardware and software has motivated the adoption of software-defined radio (SDR) as a radar platform. In an SDR, many functions traditionally implemented in hardware—such as waveform generation, filtering, and signal processing—are implemented in software, allowing the radar to be reconfigured and optimized solely through programming. With the increasing availability of commercial SDR hardware, SDR-based radar systems have become an important tool for rapid prototyping and experimental research [2]. However, SDR-based radar presents its own challenges: (1) Most commercial SDRs provide insufficient instantaneous bandwidth for high-resolution time-domain radars. (2) Software processing introduces delays compared to dedicated hardware, affecting range measurements. (3) High sample rates generate massive data volumes that challenge the data throughput constraints of host systems. (4) Phase noise and timing jitter affect coherent processing. (5) Multi-channel systems are required for accurate delay estimation and synchronization. (6) Effective implementation requires expertise spanning RF engineering, digital signal processing, embedded systems, and high-performance computing.

### 1.3. Survey of Existing SDR Radar Systems

Numerous SDR-based radar systems have been reported in the literature. Table 1 summarizes the specifications of several SDRs used in radar applications and highlights their range resolution limitations. The costs reported in Table 1 are indicative values and may vary depending on the system configuration, supplier, and time of purchase. For modular SDR platforms, such as the USRP2, USRP N210, and USRP X310, which require separate RF daughterboards, the listed price refers to the base unit and excludes the cost of the required RF daughterboard(s), which must be purchased separately. In contrast, integrated SDR platforms, such as the USRP B210, ADALM-PLUTO, and bladeRF 2.0 micro, incorporate their RF transceiver functionality on the main board and do not require separate RF daughterboards. Therefore, the reported costs are intended primarily to illustrate the relative cost differences among the considered SDR platforms rather than to represent fixed or directly comparable system prices.

#### 1.3.1. Time-Domain Approaches

An affordable impulse radar using the ADALM-Pluto was demonstrated in [3], illustrating the feasibility of compact SDR hardware for radar prototyping. However, its instantaneous bandwidth of 56 MHz limits the achievable range resolution to approximately 2.6 m, which is insufficient for many applications requiring finer precision. Similar works have been reported using USRP2 [4] and BladeRF [5] with the same limitations. Narrow-pulsed time-domain radar (e.g., impulse radar) offers a simple and fast approach to achieving high range resolution by using a very wide instantaneous bandwidth.

**Table 1:** Summary of range resolution for different SDRs used in radar applications. Reported prices are indicative and are intended primarily to highlight the relative cost differences among the considered SDR platforms.

Study	SDR	Maximum Bandwidth (MHz)	Equivalent Range Resolution in Free Space (m)	Base Unit Price (USD)
[3]	ADALM-Pluto	56	2.68	250
[6]	BladeRF 2.0 Micro	56	2.68	1300
[4]	USRP2	40	3.75	discontinued
[7]	USRP B210	56	2.68	2400
[8]	USRP N210	25	6	discontinued
[9,10,11,12,13,14,15,16]	USRP X310	160	0.94	11,000 ^1^

^1^ The present work uses UBX-160 daughterboards with the USRP X310; each daughterboard costs approximately USD 2600.

#### 1.3.2. Frequency-Domain Approaches

Step-frequency continuous-wave (SFCW) radars implemented on platforms such as the BladeRF 2.0 Micro [6] can achieve higher resolutions by sweeping through a wide frequency range, but this approach requires sequential tuning of the center frequency, which usually results in slow acquisition times that restrict real-time operation. Real-time FMCW radars have also been demonstrated using SDRs such as the USRP B210 [7] and USRP N210 (originally named NI2920) [8], but these systems are typically limited to moderate bandwidths, preventing fine range resolution. In practice, most commercial SDRs cannot generate sufficiently wide and linear chirp waveforms to support high-resolution FMCW operation.

#### 1.3.3. Summary of Limitations

Both time-domain and frequency-domain SDR radar approaches suffer from fundamental limitations. The limited instantaneous bandwidth of most SDR platforms limits the use of time-domain methods. Frequency-domain methods are limited by slow data acquisition due to sequential frequency stepping or sweeping. As a result, no existing low-cost SDR implementation meets the requirement for real-time, high-resolution radar operation.

### 1.4. Research Objectives

The original motivation for this study is rooted in an attempt to develop an SDR radar platform suitable for deployment on unmanned aerial vehicles (UAVs) for ground-penetrating radar. Time-domain radar is intended for achieving a larger penetration depth through better interference rejection which is the subject of our future work. The aim is to achieve performance comparable to dedicated radar hardware while maximizing the inherent advantages of SDR, such as low power consumption, reconfigurability, and cost-effectiveness.

To enable rapid implementation of impulse radar while maintaining high range resolution, this work uses a USRP X310 equipped with a UBX-160 daughterboard, which supports up to 160 MHz of instantaneous RF bandwidth. Using this bandwidth necessitates high-speed analog-to-digital conversion, efficient host-to-SDR interfaces, and meticulous system configuration. Despite these capabilities, relatively few published studies have employed the X310 for wideband time-domain radar.

### 1.5. Experience with GNU Radio

Dedicated software frameworks have been developed to support SDR implementation on general-purpose PCs. Among these, GNU Radio is the most widely used open-source platform and is supported by a large developer community.

Initial exploration of GNU Radio as the SDR development framework showed that its graphical interface, GNU Radio Companion (GRC), enables users to build signal-processing chains using block diagrams, improving accessibility and ease of use. However, several limitations were identified that restricted radar performance.

First, the abstraction layers and graphical execution model introduce significant computational overhead and processing delays. Consequently, the high sample rates required for wideband radar exceeded GNU Radio’s real-time processing capabilities, limiting the sample rate to 25 MS/s due to the particular CPU and network card used in the host computer. Additionally, unpredictable latency complicates timing-critical radar operations; however, at the time of writing this paper, proper blocks do not exist to extract USRP’s timestamps directly. The automatically generated Python code of the flowgraph must be manually modified to access timestamps, resulting in a more cumbersome development workflow. Therefore, sustaining accurate timing between transmit and receive channels proved difficult within the framework.

These limitations are particularly problematic for applications that require very high sample rates and low latency, such as wideband real-time radar.

### 1.6. Challenges with USRP X310 Implementation

Most existing implementations rely on frequency-domain techniques such as dual [9] and multiple [10] frequency continuous wave (CW), random CW [11], FMCW [11,12], SF-LFM [13], FMiCW [14], SFCW [15], and OFDM [16] which synthesize wide bandwidth by sweeping the center frequency rather than true wideband sampling. While these methods can achieve high resolution, they are inherently slower and more complex than impulse-based approaches.

Implementing wideband impulse radar on the X310 poses considerable challenges, requiring significant expertise across multiple domains including: (1) C++ programming for real-time data handling, (2) hardware understanding of X310’s subsystems including FPGA, ADC/DAC, and clocking, (3) efficient data processing of continuous high-rate data streams, (4) high-rate data interfaces (i.e., USB3 and 10 Gb Ethernet) for sustained throughput, and (5) system configuration for optimizing network buffer sizes, ring buffers, and CPU governors.

Because commercial SDRs are not specifically designed for radar applications, custom solutions were developed to optimize the platform for the requirements of this research.

Moreover, in nearly all reported cases, the X310’s full instantaneous bandwidth has not been utilized, either due to hardware limitations or a suboptimal host configuration. For example, in [12], the sampling rate was restricted to 100 MHz due to insufficient memory to handle the resulting data volume.

In addition, many publications do not provide the detailed hardware specifications and host-side configuration parameters required to sustain high-rate streaming, making their results difficult to reproduce. For example, a time-domain radar using the X310 was presented in [17]; however, that work focuses primarily on interference mitigation in cognitive radar systems and does not describe the system configuration or implementation details required for wideband, real-time operation.

Furthermore, many existing SDR-based radar systems rely on graphical programming environments, which introduce significant computational overhead and limit achievable real-time performance at high sample rates. An open-source software framework for ice-penetrating radar was reported in [15] and deployed on Ettus platforms, including the X310 and B205mini-i, using C++ and Python. While that work provides useful guidance on compilation and software configuration, it is designed for frequency-domain radar, and does not address the hardware and streaming requirements necessary for wideband impulse operation.

### 1.7. Single-Channel Implementation

Most SDR-based radars employ two RF channels to synchronize transmission and reception. For the UBX-160, the transmitter and receiver use separate local oscillators, so a second channel is commonly used to capture a reference copy of the transmitted signal. Because the two daughterboards on the X310 are phase-coherent, delays can then be estimated between the received echoes and the reference signal [18]. Alternatively, some studies have used a single channel together with a known reference target for calibration [4,5].

This work demonstrates that the built-in timestamping capabilities of the USRP, combined with calibration against a known reference target, enable accurate range estimation using only a single RF channel, thereby eliminating the need for a dedicated reference path.

### 1.8. Contributions

The main contributions of this work are as follows:

1. Flexible architecture: An SDR-based radar framework that can be later extended to multiple radar modalities (impulse, FMCW, SFCW).
2. Real-time high resolution: Achieved through wide instantaneous bandwidth (160 MHz) and optimized high-data rate streaming. To the best of the authors’ knowledge, this is the first published time-domain radar implemented with USRP X310 at 200 MS/s data rate.
3. Single-channel implementation: Accurate ranging using timestamping and calibration without a dedicated reference channel.
4. Quantified performance bounds: Experimental characterization of the platform’s deterministic processing delay (approximately 620 ns) and stochastic inter-scan timing jitter (mean 8.8 ns, standard deviation 26.1 ns), together with a controlled two-path experiment establishing the practical round-trip range resolution (2 m) relative to the theoretical bandwidth-limited value (0.94 m).
5. Low-level C++ implementation: Maximized efficiency through direct USRP Hardware Driver (UHD) API access, bypassing graphical frameworks.
6. Reproducible guidelines: Detailed hardware and configuration parameters for the research community.

### 1.9. Paper Organization

The remainder of this paper is organized as follows: Section 2 describes the system hardware and software requirements; Section 3 details the necessary configuration steps; Section 4 presents the C++ radar implementation; Section 5 discusses experiments and results; and Section 6 concludes the paper, outlining possible improvements.

## 2. System Requirements

The performance of an SDR-based radar system depends not only on the radio hardware but also on the capabilities of the host computer and the efficiency of the data interface between them. In wideband, real-time radar applications, if the SDR does not fully utilize its bandwidth, the host computer and network subsystems are likely to be the limiting factors rather than the SDR itself. Identifying appropriate hardware and software was therefore a critical aspect of this research. This section summarizes the components used in the system and provides a reference configuration for researchers attempting to reproduce similar performance.

### 2.1. USRP X310 with UBX-160 Daughterboard

**Figure 1:**
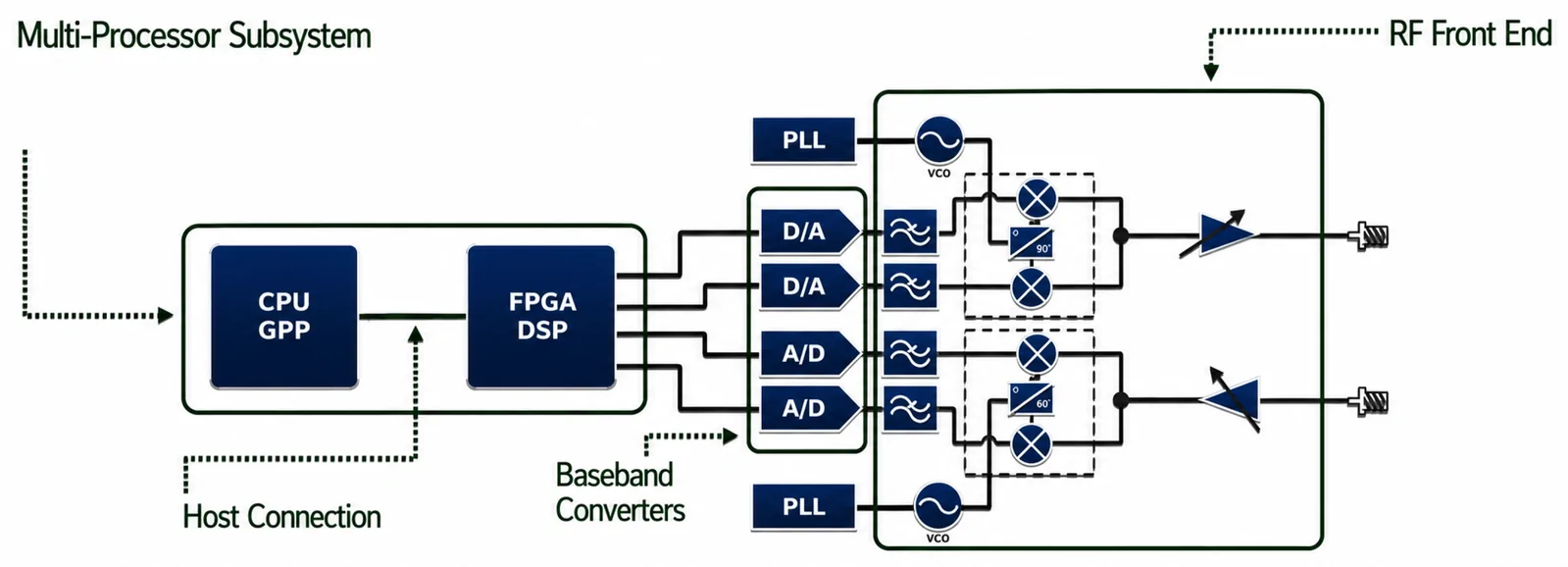
Simplified block diagram of USRP X310 [19].

The Universal Software Radio Peripheral (USRP), developed by Ettus Research, is a family of SDR devices designed for high-performance and flexible radio applications. The USRP X310 is especially suitable for wideband radar because it supports up to 200 MS/s streaming bandwidth, much broader than most comparable SDRs, and provides two independent 10-Gigabit Ethernet (10 GbE) interfaces for high-bandwidth data transfer. In addition, it features a powerful onboard FPGA that can perform substantial real-time signal processing. Figure 1 shows a simplified block diagram of the USRP X310.

The X310 supports two full-duplex RF channels via interchangeable daughterboards. In this work, a UBX-160 daughterboard is used, providing frequency coverage from 10 MHz to 6 GHz and an instantaneous RF bandwidth of up to 160 MHz. This wide bandwidth enables fine range resolution within time-domain radar applications. Unlike most previously published implementations, only a single RF channel is used here. As explained in Section 1.7, the USRP’s built-in timestamping capabilities are used to estimate signal time-of-arrival without requiring a second synchronized receive channel.

### 2.2. Dual 10-Gigabit Ethernet Interface

The network interface between the host PC and the USRP X310 is one of the most critical components for achieving high sample rates. To support 200 MS/s data streaming, two independent 10-Gigabit Ethernet links are required. In this work, a dual-port 10 GbE network interface card (Intel Ethernet Controller X550-T) with a maximum “ring buffer” size of 32,768 Bytes is used, together with two SFP+-to-RJ45 adapters and two Category-6 10 GbE cables. A ring buffer is a circular buffer that holds descriptors or pointers to socket kernel buffers containing the packet data. It is essential to adjust the ring buffer size to prevent packet drops and improve network performance. A smaller ring buffer size can lead to underflow or overflow. While this setup successfully handles 100 pulses (each 99.8 us long) and 19,960 samples per pulse, slower interfaces or using only one 10 GbE interface result in mass underflow and overflow of samples, corrupting the waveform. Newer network cards, such as Intel X710, Mellanox ConnectX-4, and Mellanox ConnectX-5, provide larger ring buffers which should increase reliable throughput and data rates.

On laptops and computers which typically lack a 10 GbE port, a Thunderbolt 3-to-10 GbE adapter, such as the Sonnet Solo 10G, can be used to provide the required bandwidth. Both approaches were tested and found to be functional for high-rate streaming.

### 2.3. Operating System

Ubuntu 22.04 is selected as the host operating system because the manufacturer recommends it for stable X310 operation. Using the officially supported operating system simplifies troubleshooting and guarantees compatibility with the USRP Hardware Driver (UHD). Linux-based systems are generally preferred for SDR applications, since most open-source SDR frameworks and performance-tuning tools are developed and optimized for Linux. Attempts to use Windows 11 resulted in persistent dependency and installation issues, making it unsuitable for reliable high-rate SDR operation in this context.

### 2.4. Host Computer

A few different host computers were used in this work. The first is equipped with a 12th-generation Intel Core i7-12700KF processor with eight performance cores and four efficiency cores, for a total of 12 cores and 20 threads. The processor supports clock speeds up to 5 GHz. For stable real-time performance, the computational workload can be pinned to the performance cores, as will be discussed in Section 3. High clock speeds are particularly important for handling high-rate data streams without buffer underruns. This system is equipped with 64 GB of RAM and a 2 TB solid-state drive, ensuring sufficient memory and storage for high-volume data acquisition and processing.

Because the initial system was a bulky desktop, a laptop (ASUS Zenbook Duo) was subsequently tested for portability. The configuration details are summarized in Table 2. As the laptop does not support 10GE, an alternative network configuration employing two Thunderbolt 3-to-10 GbE adapters was implemented. This system was nearly successful, with only occasional failures in writing samples to memory.

Finally, a lightweight mini-PC (Minisforum MS-01) with the configurations shown in Table 2 was used. This system provides two built-in 10 GbE connections, although the maximum ring buffer size remains limited (8160) for the application. However, it offers a PCIe slot which was used to install a network interface card with a maximum ring buffer size of 32,768 (Intel Ethernet Controller X550-T). This configuration proved successful and is recommended for similar implementations.

### 2.5. Antennas

The choice of antennas depends on the intended application and operating frequency range. In this work, two commercially available Vivaldi antennas with a bandwidth of 600–6000 MHz are used. The antennas are connected to the SDR via SMA cables and are spaced 45 cm apart.

### 2.6. Software Environment

USRP devices are programmed through the USRP Hardware Driver (UHD), an open-source framework that provides a unified API for device configuration, timing, synchronization, and data streaming. UHD supports development in C++, Python, and GNU Radio.

GNU Radio Companion (GRC) enables rapid prototyping through graphical flowgraphs, while Python provides convenient access to the UHD Application Programming Interface (API) and scientific computing libraries such as NumPy and SciPy. However, both approaches introduce additional abstraction layers that can limit performance at very high data rates. The maximum achievable bandwidth in the same setup was 25 MS/s. Also, the GNU Radio Companion blocks at the time of this work do not provide timestamping for USRPs.

Accordingly, the radar system in this work is implemented using the UHD C++ API at a low level. This approach delivers direct control over buffers, timing, and hardware configuration, resulting in lower latency and higher throughput. While implementing at 50 MS/s was not feasible with GNU Radio Companion during testing, the C++ implementation operates reliably at 200 MS/s. Furthermore, the C++ framework also enables modular radar design, facilitating adaptation to different radar architectures.

## 3. Preparing the System

Reliable operation of the USRP X310 at high data rates and wide bandwidth requires careful hardware and software preparation. While general guidelines in the UHD and USRP manuals [20] are helpful for communication systems, integrating all necessary steps for wide-bandwidth radar is challenging. Radar systems require precise real-time device control, which is more stringent than typical communication systems, where nanosecond delays are less critical. When properly configured, the USRP can achieve this level of control. At high data rates, sample underflow and overflow can occur, invalidating measurement data. To prevent these issues, software configurations must be optimized for high data rates. This section consolidates the essential procedures for configuring the system, focusing mainly on software.

### 3.1. Hardware Setup

The UBX-160 daughterboard is first installed in the X310 chassis. The device is initially connected to the host computer using a standard 1-Gigabit Ethernet interface. A detailed installation and connection procedure is available in the manufacturer’s documentation [21]. Successful connectivity is verified by pinging the device IP address (192.168.10.2 by default) from Terminal (Ubuntu’s command-line interface).

### 3.2. UHD Installation

The USRP Hardware Driver (UHD) is installed from source to ensure compatibility and optimal performance. For Ubuntu 22.04, the required dependencies are installed according to the official Ettus documentation [22]. UHD is then cloned, built, and installed using the following procedure in Terminal (Listings 1–3).

**Listing 1.** UHD installation (bash).

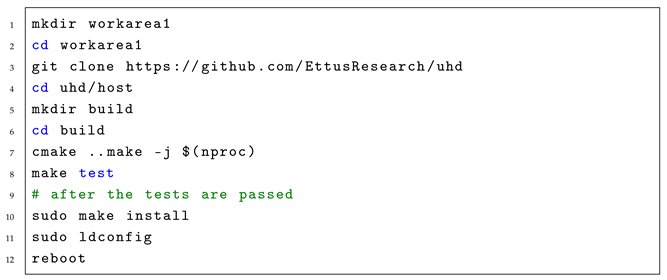



After rebooting, the installation can be verified using one of the following:

**Listing 2.** Installation verification (bash).

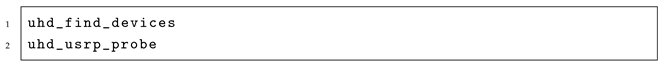



If required, the FPGA image is updated using

**Listing 3.** Image update.

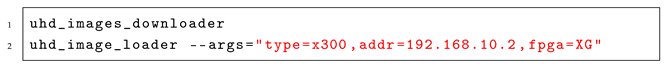



### 3.3. Upgrading to Dual 10-Gigabit Ethernet

To enable streaming at the maximum supported data rate, the default 1-Gigabit Ethernet interface is replaced with a dual 10 GbE configuration requiring a two-port 10-GbE network interface card, two SFP+-to-RJ45 adapters, and two 10 GbE Ethernet cables. Alternatively, a Thunderbolt 3-to-10 GbE adapter can be used. Selecting the “XG” FPGA image as in Listing 3 is necessary to provide dual-10 GbE operation. Following this step, the IP addresses for the two Ethernet ports will be set to static values, 192.168.30.2 and 192.168.40.2.

### 3.4. Network Configuration

To ensure high-throughput and low-latency data streaming, the host network configuration must be optimized. The following commands increase the maximum transmission unit (MTU) to enable jumbo frames, enlarge kernel socket buffers, and maximize network interface ring buffers [23]. An MTU value of 9000 is specifically required. For read and write socket buffers, a value of 33,554,432 (=2^25^) Bytes was successful in this work.

These optimizations reduce packet loss and improve streaming reliability. Although frameworks such as DPDK and the use of huge pages can further improve throughput, they were not required to achieve the data rates reported in this work.

### 3.5. CPU Tuning

Modern CPUs dynamically adjust their clock speed to regulate power consumption and performance. For real-time radar operation, it is advantageous to execute the streaming and processing threads on the performance cores; otherwise, underflow can occur when transmitting at high sample rates. The following command (Listings 4 and 5) sets the CPU governor to “performance” mode for each core.

**Listing 4.** Network configuration (bash).

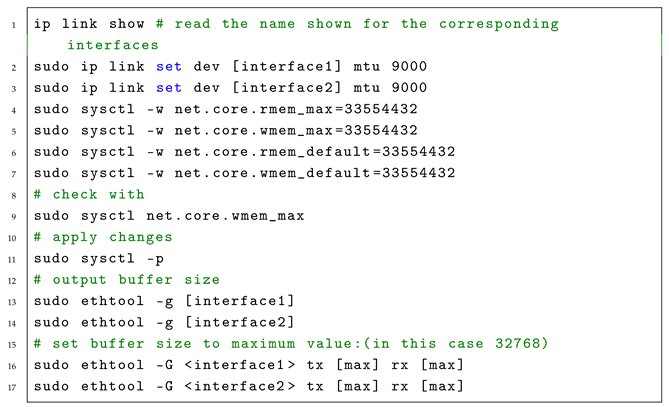



**Listing 5.** CPU tuning (bash).

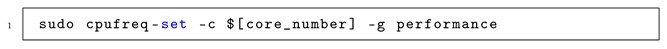



Although “performance” mode increases power consumption, it is recommended for prioritizing the maximum performance.

### 3.6. Performance Test

Before running the radar application, the system is validated using UHD benchmark tools. The “benchmark_rate” example in the UHD directory [24] is used to verify that the Ethernet interface can sustain the desired transmit and receive rates without flow control errors (e.g., dropped samples, overruns, or underruns). For example, the following command (Listing 6) checks for flow-control errors at the transmit and receive rate of 10 MHz for 300 s.

**Listing 6.** Benchmark test (bash).

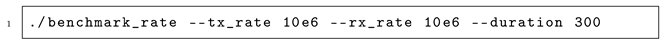



The “benchmark_rate” utility only assesses whether the Ethernet interface can reliably sustain the requested data rate. It does not take into account any load to CPU or disk I/O. If operating an application at those rates results in any flow control errors, then either the application must be optimized or the CPU must be upgraded.

Additional example programs, such as “*tx_samples*” for transmitting, “*rx_samples_from_file*” for receiving, and “*txrx_loopback_to_file*” for both transmission and reception, are executed at 200 MS/s to ensure stable operation.

Once these tests are completed without errors, the system is ready for custom radar software.

## 4. Radar Programming

As discussed in Section 1, the UHD C++ API provides fine-grained control over memory management, timing, and hardware interaction, enabling higher throughput and lower latency than high-level frameworks. In this section, the implementation of a time-domain impulse radar developed for this work is discussed. The program is based on the UHD example “txrx_loopback_to_file.cpp”, with major modifications to support timestamping, arbitrary waveform transmission, and radar-specific post-processing. While the complete source code tailored for this specific application is available at [25], the key components are explained here to provide a wider picture of radar operation.

**Figure 2:**
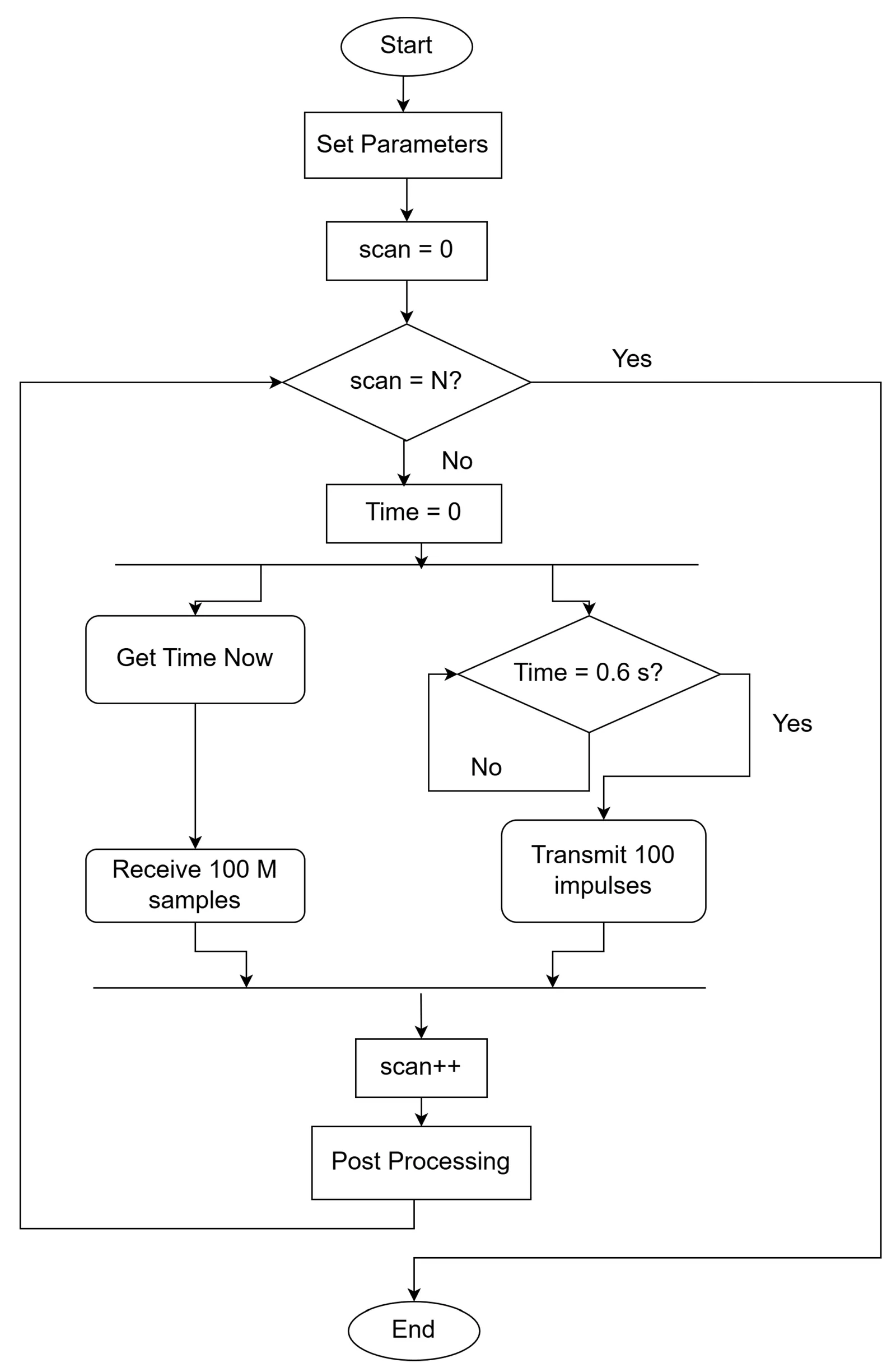
Flowchart of C++ program.

The radar system is designed to perform multiple scans, estimating the target location at each. The algorithm is shown in Figure 2. Each scan consists of a transmission and a reception process executed concurrently using multithreading. This allows the transmitter and receiver to operate in parallel, exploiting the host PC’s multi-core architecture.

**Figure 3:**
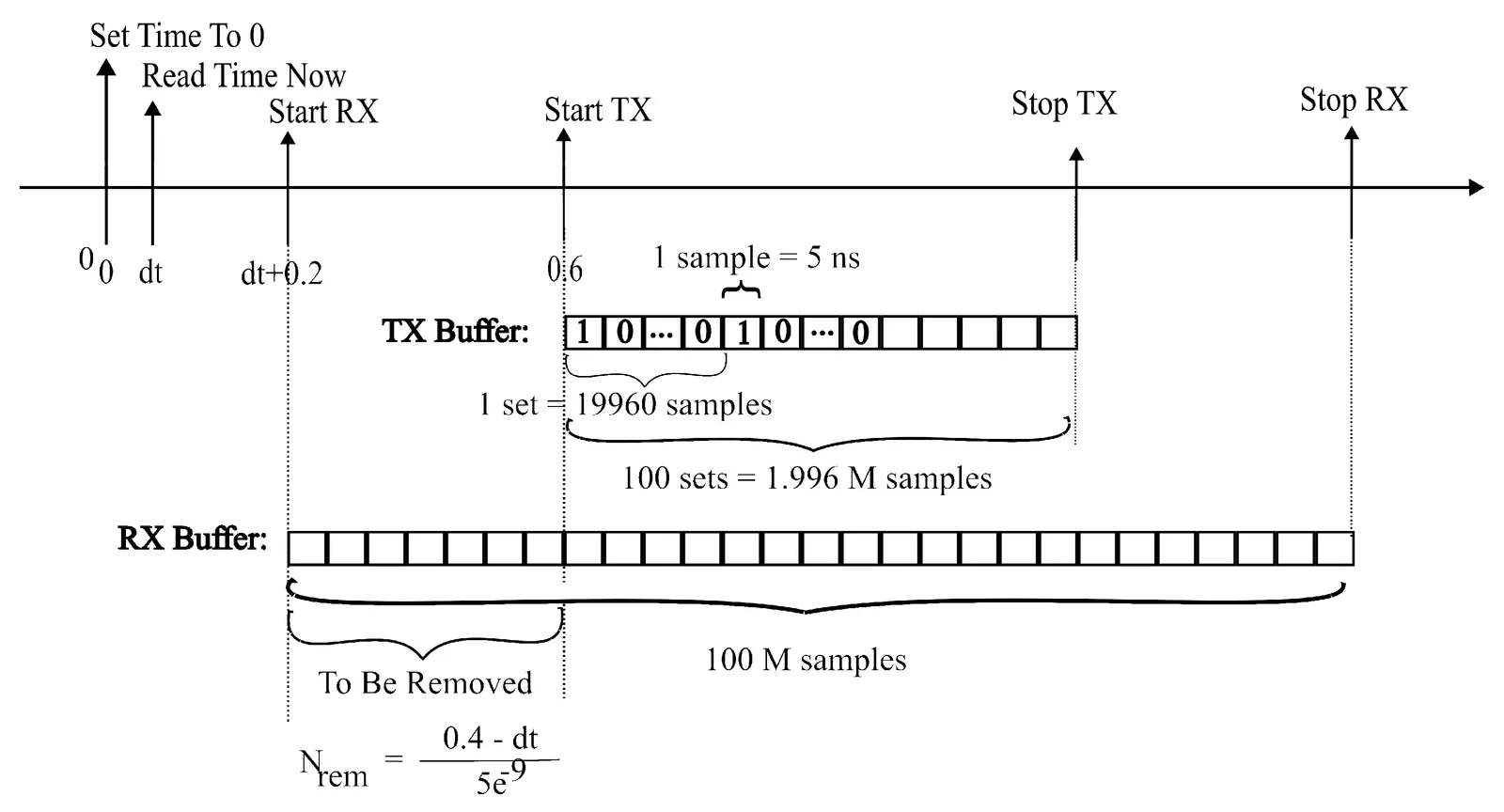
Timeline of each scan.

As shown in Figure 3, in each scan, the transmission thread emits a hundred 5-ns-wide pulses separated by 99.8 µs, corresponding to 19,960 samples at a sampling rate of 200 MS/s (5 ns sampling interval). In parallel, the reception thread continuously acquires 100 million samples. To ensure that no transmitted signal is missed, reception begins slightly before transmission. After both threads are completed, the recorded data are processed to extract target’s range.

### 4.1. Waveform Generation

The UHD example programs provide only simple predefined waveforms, such as constant, sinusoidal, rectangular, or ramp signals, which are insufficient for radar operation. Radar systems usually use different waveforms, such as chirps, stepped-frequency signals, or specific pulse shapes, for different purposes. To develop the most flexible program for a multi-architecture radar system, it is best to use an externally generated waveform and import it as a file. Therefore, the code was extended to read an arbitrary waveform from a text file. This approach allows waveforms to be generated offline and loaded during runtime, reducing computational overhead and permitting rapid reconfiguration. Once the data is read from the file, it is assigned to the transmit signal within the main program.

Although this work focuses on impulse radar, the same mechanism allows chirps or multitone signals to be used for other radar architectures. Care must be taken to ensure that waveform parameters, including sample rate and samples per symbol, are compatible with the transmit and receive rates to preserve the intended signal shape. Otherwise, it will be either scaled in time or distorted according to the Nyquist criterion.

In the present implementation, the transmitted waveform consists of a single sample having amplitude 1, corresponding to an approximately 5-ns pulse at 200 MS/s—a good approximation to an ideal impulse for wideband radar operation. During the transmission thread, this file is read and transmitted 100 times at each scan.

### 4.2. Timestamping

As discussed in Section 1.7, only one RF channel is used; therefore, accurate timestamping is essential for determining the time of arrival of the received echoes. UHD timed commands are used to record the precise timing of transmission and reception. To ensure no data is lost, reception must always begin before transmission.

First, the USRP time is set to zero. Then, transmission and reception streams are programmed. Right before starting reception, the timestamp is stored in the variable “*dt*”. Then, the receiver waits 0.2 s for the RX local oscillators to stabilize at their designated frequencies. Therefore, the receiving time is 0.2 s + *dt*. The transmission is also scheduled to occur 0.6 s after the time origin. Empirically, this 0.6 s delay ensures that transmission occurs after reception (0.2 + *dt*).

For time alignment, any portion of the received signal captured before the start of transmission is discarded. This corresponds to a time interval of 0.4 s − *dt*. Samples in this interval are dropped. However, the remaining portion of the signal still does not represent the true target range, as it includes inevitable internal processing lags. For range measurement purposes, a known reference target can be used for calibration, as in [4,5].

Executing the following commands (Listing 7) immediately before enabling the receiver stream finds the time *dt*, which is used to estimate the transmission–reception time difference as explained above.

**Listing 7.** Receiver timestamp (C++).

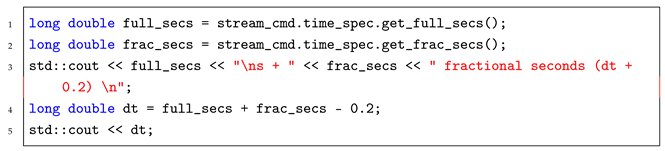



### 4.3. Post-Processing

A post-processing function is defined and invoked to extract the desired part of the received signal and plot real-time figures. For an intuitive graphical demonstration, a two-dimensional (2-D) plot is generated showing the target’s location at each scan time. This approach provides real-time monitoring in the final application, as well as performance evaluation and troubleshooting during development, allowing identification of any mistakes before continuing the measurement.

As explained earlier, the radar system is designed to perform multiple scans, estimating the target location at each. During each scan, a transmission and a reception thread are executed simultaneously. The transmission thread emits 100 impulses separated by 99.8 μs, including 19,960 samples, and the reception thread acquires 100 million samples before stopping. All received data in buffers are stored to memory. Since using the C++ insert() method is too slow for this amount of data, a raw byte-by-byte memory copy is implemented using memcpy() to decrease the time of each run by several seconds. After both threads complete execution, a post-processing stage is performed. During post-processing, timestamps are used to discard the initial portion of the received data captured before transmission began, as explained in Section 4.2. As a result, the processed reception data contains responses corresponding to the 100 transmitted impulses. Ideally, for a single target reflection, it should look like an impulse train.

**Figure 4:**
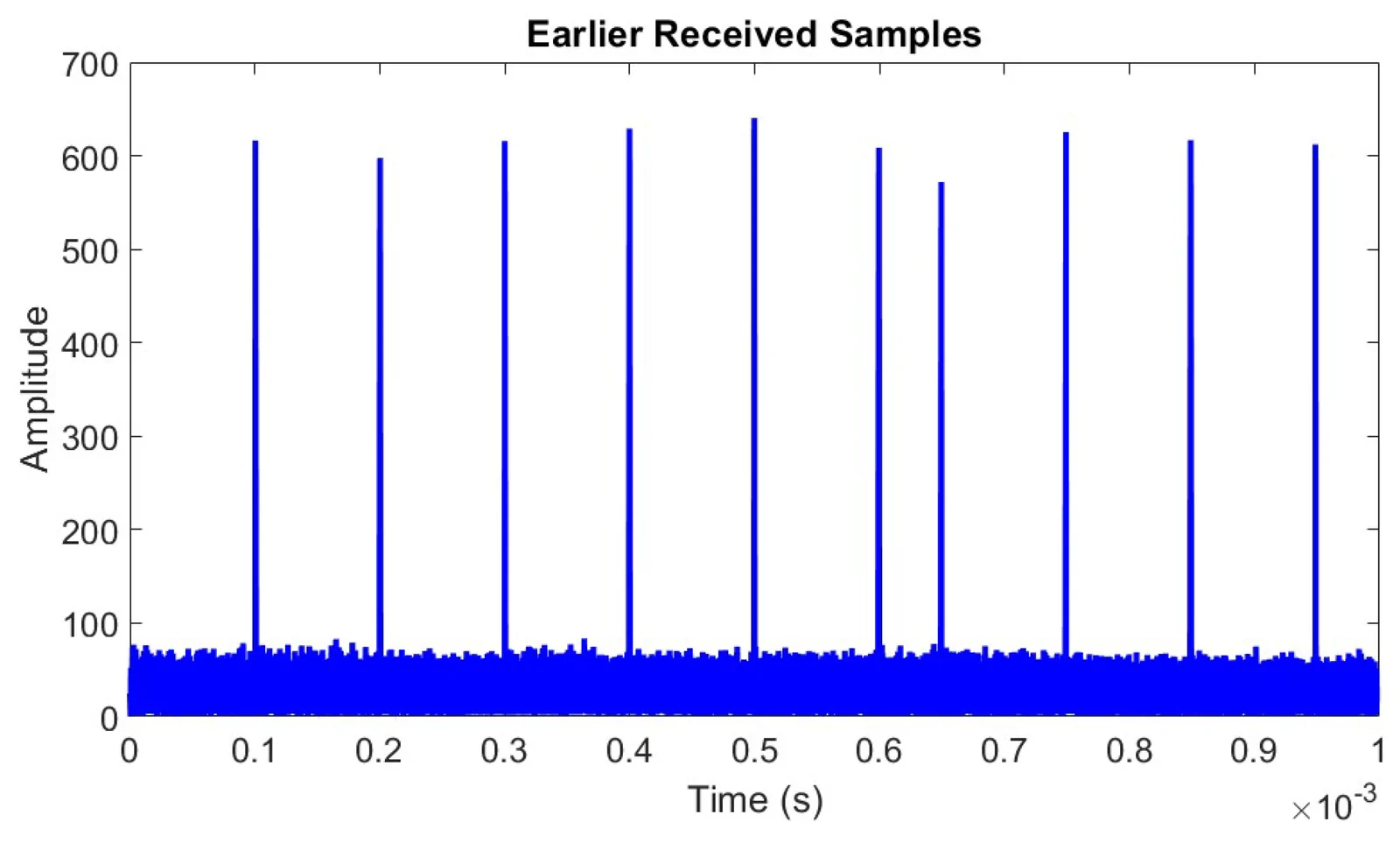
First received impulses.

As shown in Figure 4, the initial impulse responses exhibit inconsistency. First sample, and consequently all other samples are delayed by 620 ns, showing a processing delay which is almost consistent through the measurements. First impulse is also very low in amplitude. In addition, the seventh impulse response contains only 9980 samples, exactly half of the expected 19,960 samples. The exact cause of this isolated event could not be conclusively determined. However, it may be associated with transient streaming or acquisition behavior during high-rate data transfer, including possible buffer or timing effects in the SDR data path. Since this behavior occurs only once during the acquisition and does not repeat in the subsequent samples, the affected response is excluded from further processing. The first 10 impulse responses are therefore discarded to avoid potential initialization and acquisition transients, and the subsequent 50 responses are retained for analysis.

**Figure 5:**
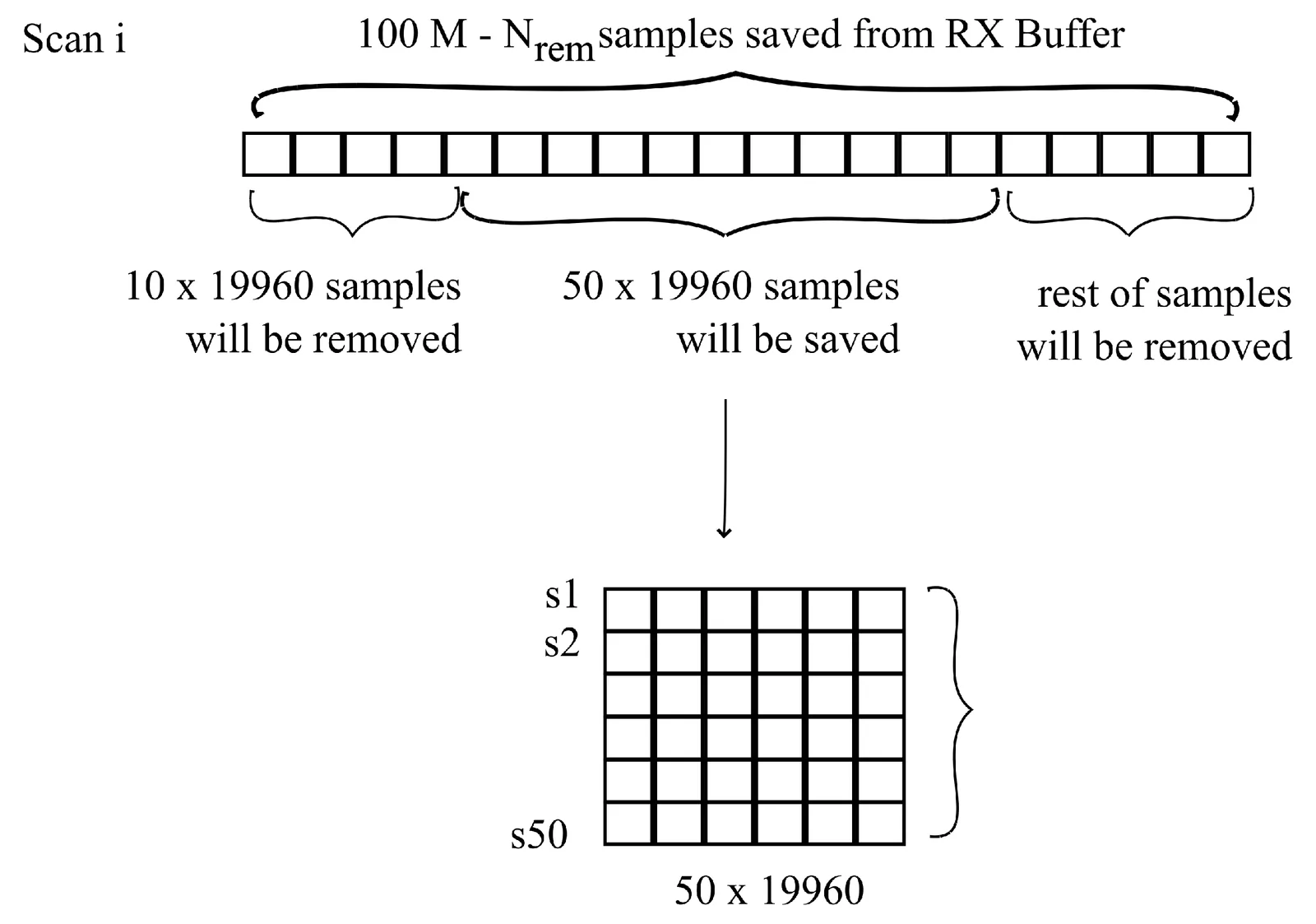
Post-processing algorithm.

These 50 truncated responses are stored to disk for further post-processing. Binary write is used for faster writing to disk. These responses are stored in a matrix *S* of size 50 × 19,960, where each row $s_{i}\left(i=1,\ldots ,50\right)$ represents a single impulse response. Figure 5 shows how this data is handled during post-processing for each scan. Further post-processing and visualization is explained in Section 5.

### 4.4. Compilation and Running

The source code is converted to an executable file using the g++ compiler from Terminal as in line 1 of Listing 8. Necessary headers (only if they are not installed in the default directory) and libraries used in the C++ program should be properly included using -I and -L flags similar to lines 2 to 4 in Listing 8. Libraries such as UHD, Python (used for post-processing), and Boost (a collection of libraries used by UHD) must also be linked as in line 5 and 6 of Listing 8. The exact compilation command may vary depending on the system configuration and library installation directories. For example, in this work, the compilation command was as follows:
**Listing 8.** Compilation (bash).
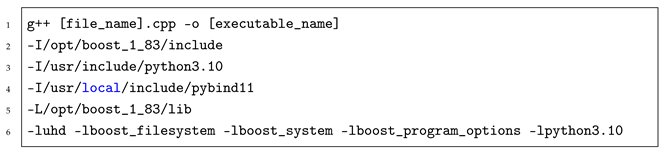


Once compiled, the executable is run with command-line arguments to specify the frequency, gain, sample rate, and the number of received samples, in the same manner as the UHD example program, “txrx_loopback_to_file”. An example command (Listing 9) is shown below:
**Listing 9.** Execution (bash).
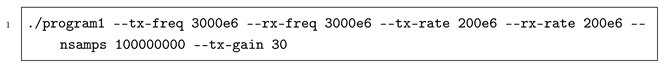


## 5. Experiments and Results

Before moving on to the radar measurements, the output power of the UBX-160 transmit port and the daughterboard’s bandwidth at different center frequencies are evaluated. Although the UBX-160 is known to have a 160 MHz bandwidth, according to the manufacturer, the receiver path has 84 MHz of bandwidth for center frequencies from 10 MHz to 500 MHz [26]. In this work, an arbitrary center frequency of 2 GHz is chosen which supports a 160-MHz bandwidth. A simple loopback test is also conducted to verify the processing method and examine the range resolution.

### 5.1. Signal Amplitude and Output Power

The transmitted power of each frequency tone (including cable losses) is measured and shown in Table 3 at different center frequencies. A constant waveform having an amplitude of 0.3 and a transmission gain of 0 dB is used. In UHD, all signals are normalized between −1 and 1 values. The amplitude of 0.3 is chosen to ensure linear operation up to 30 dB transmit gain at 2 GHz (operating frequency for this work). As frequency increases, the output power of USRP generally decreases.

### 5.2. Loopback Resolution Test

**Figure 6:**
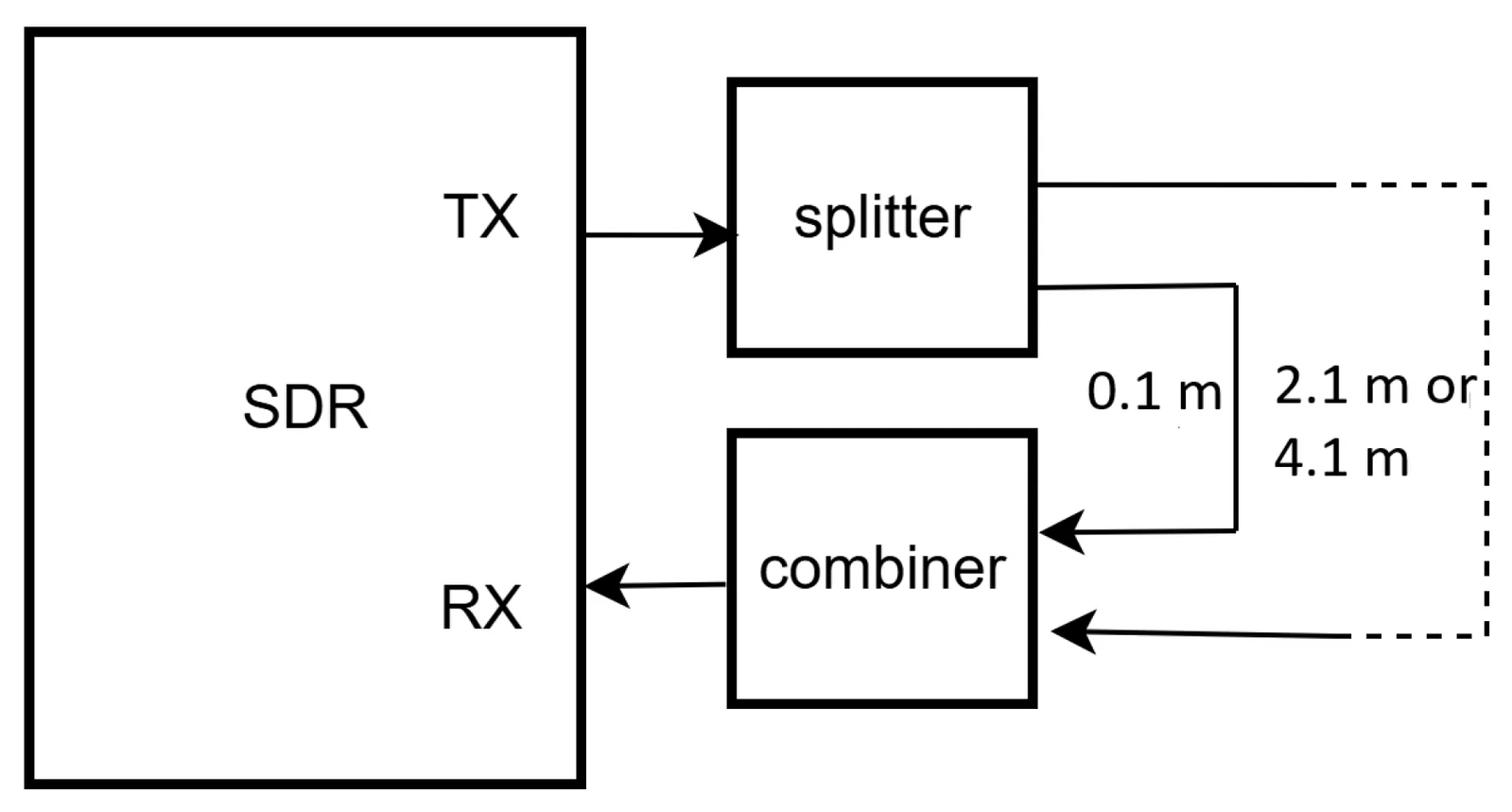
Measurement setup for two-path loopback test.

A loopback test was conducted to validate the received signal and experimentally characterize the system’s range resolution. As shown in Figure 6, the setup consisted of two propagation paths: a 0.1 m reference path and a longer path of either 2.1 m or 4.1 m. The additional 2 m or 4 m propagation length was implemented using air-filled coaxial cables.

**Figure 7:**
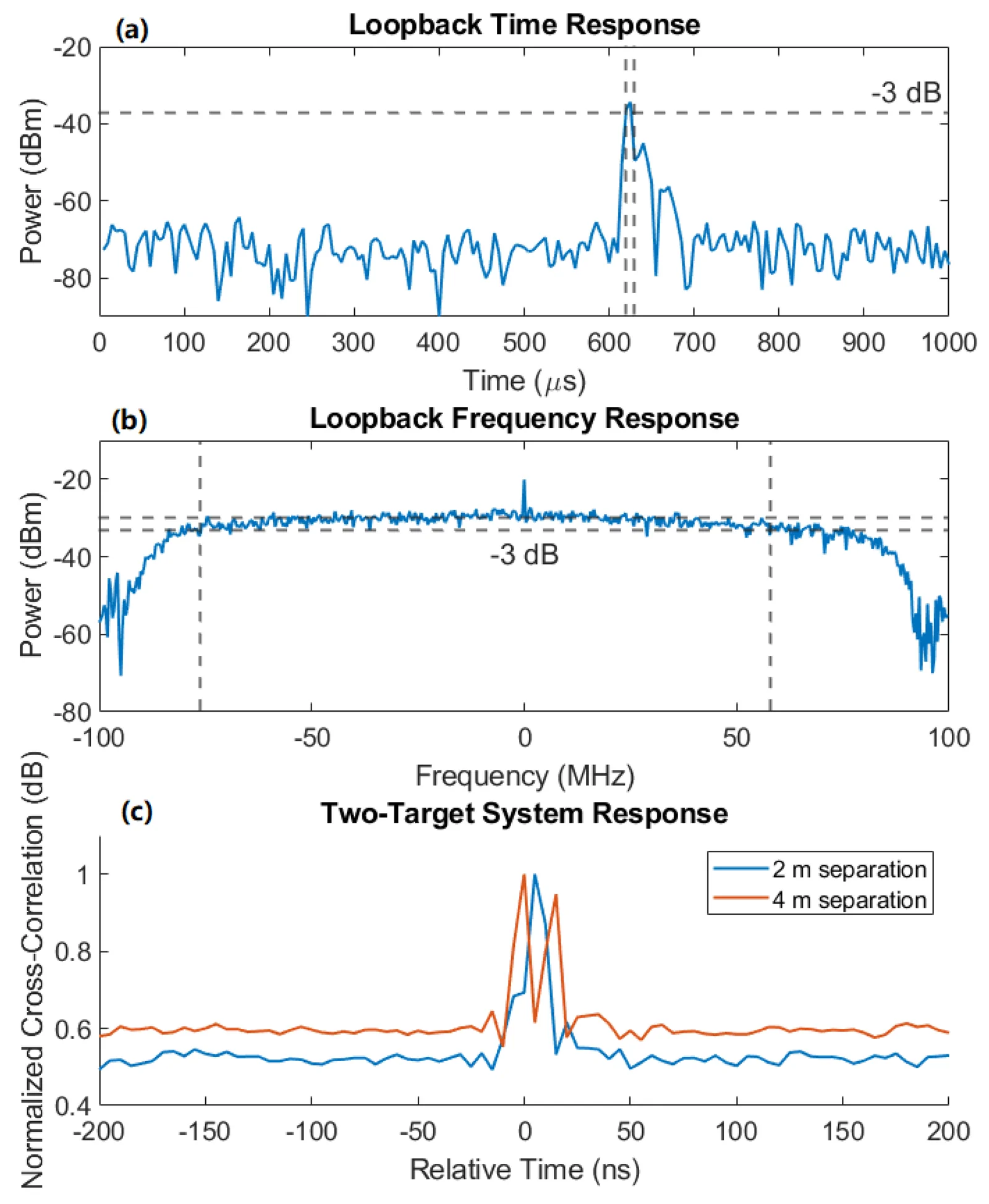
Two-cable system impulse response.

The long path was first disconnected to characterize the system response of the 0.1 m reference path, including the effects of the cables and power splitters. A pulse with a digital duration of 5 ns was transmitted through the system. The measured time-domain response (i.e., the received signal) is shown in Figure 7a. The received pulse is broader than the nominal digital pulse because of the limited RF bandwidth of the system and is further distorted by the frequency response of the power splitters. The peak of the received response occurs at sample 125, corresponding to 625 ns. This delay is attributed to the transmit and receive processing chains, including FPGA processing, and is therefore used as the reference system delay for determining the propagation delay through the longer path [4,5]. This is 5 ns later than the reference delay measured in Figure 4. This misalignment in the reference delay is later discussed in Section 5.3.

The measured impulse response has its maximum at sample 125. Sample 124 remains slightly above the −3 dB half-power level, whereas samples 123 and 126 are approximately 13 dB below the peak. Thus, the half-power crossing points occur between the available discrete samples rather than exactly at a sample location. With a sampling interval of 5 ns, the left crossing occurs between samples 123 and 124, while the right crossing occurs between samples 125 and 126. Since the right-hand crossing is closer to sample 125 than to sample 126, the measured half-power main-lobe width is slightly greater than 5 ns. This measurement provides an experimental characterization of the temporal width of the received impulse response.

Figure 7b shows the corresponding frequency response obtained from the Fourier transform of the received signal. A pronounced peak is observed at DC in the measured frequency response. This isolated component is attributed to a DC/baseband artifact of the measurement rather than to the RF passband response of the system. Therefore, the DC component was excluded when determining the effective RF bandwidth. The 3-dB bandwidth was calculated relative to the approximately flat passband level away from DC, which more accurately represents the usable RF response of the system. The measured frequency response of the configured system has a half-power bandwidth of 133 MHz, slightly less than the daughterboard’s nominal bandwidth.

For a conventional pulsed radar, the range resolution δ is approximately related to the pulse duration τ with δ=ντ/2, where ν is the propagation velocity of the wave in the medium. Equivalently, the same relationship can be expressed in terms of the transmitted bandwidth *B* with δ=ν/2B. Although with a range-bin spacing (i.e., sampling interval) of 5 ns, a pulse duration of 5 ns can be achieved, the RF front-end in USRP X310 is limited to 160 MHz, resulting in a theoretical range resolution of approximately 0.94 m. This value represents the minimum separation between two ideal point targets that can be distinguished under ideal conditions.

In practical systems, the measured range resolution is generally lower than the theoretical value due to nonideal effects, including phase noise, frequency-step errors, timing instability, antenna bandwidth limitations, multipath propagation, and residual calibration errors. Experimental evaluation using targets at known separations is required to quantify the practical resolution of the implemented radar system.

To evaluate the system’s range resolution, the long path was then connected using either a 2 m or 4 m cable extension. The responses of the two-path configurations were cross-correlated with the single-path reference response, and the delay corresponding to the maximum cross-correlation was used to estimate the range difference between the two targets. The results are shown in Figure 7c. For the 2 m path difference, only a single peak is observed at 5 ns. Ideally, two peaks should appear: one at 0 ns and a second at approximately 6.7 ns. However, the two responses overlap and cannot be resolved. In contrast, the 4 m path difference produces two distinct peaks at approximately 0 ns and 15 ns, in reasonable agreement with the theoretical delays of 0 ns and 13.3 ns. These results demonstrate that a one-way propagation distance difference of 4 m can be resolved by the proposed system.

It should be noted that this experiment evaluates one-way propagation distance, whereas the resolutions reported in Table 1 correspond to round-trip radar measurements. Consequently, the 2 m and 4 m one-way path differences are equivalent to round-trip target separations of 1 m and 2 m, respectively. Although Table 1 predicts a theoretical range resolution of 0.94 m, the experimental results show that two targets separated by an equivalent distance of 1 m cannot be reliably resolved in practice. A practical range resolution of 2 m is achieved in this experiment with the maximum available range-bin spacing of the USRP X310 (i.e., 200 MS/s).

### 5.3. Radar Measurements

**Figure 8:**
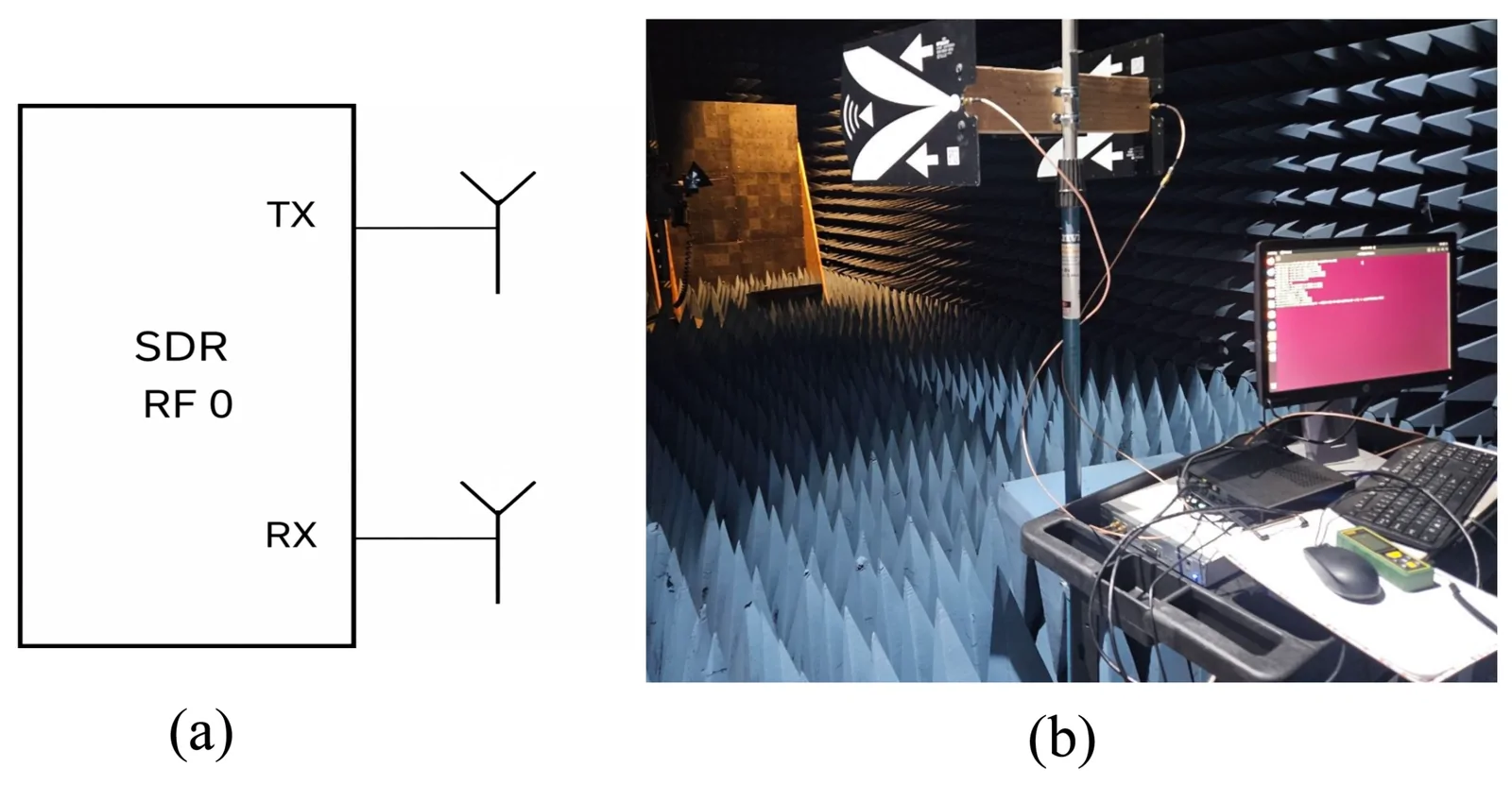
Radar measurement setup: (**a**) system schematic; (**b**) system in the low-noise environment for minimizing ambient effects. Two Vivaldi antennas are used with vertical polarization and 45 cm separation.

A range measurement radar is implemented in this work to evaluate the X310’s radar capabilities. Figure 8 shows the measurement setup. The host computer and X310 are loaded on a cart. Two Vivaldi antennas (10 dB gain at 3 GHz) are connected to the TX and RX ports of Channel 0, where the daughterboard is installed. The antennas are parallel and V-polarized, separated by 45 cm, and the cart is placed inside a low-noise environment to isolate ambient effects and confirm proper system operation. A 75 cm × 75 cm aluminum sheet serves as a moving target changing location from close to the antenna to 6 m away and back.

Sampling is done with 200 MS/s for both transmission and reception. The center frequency is set to 2 GHz, and the TX gain is 30 dB. For five repetitions of this measurement, no overrun, underrun, or dropped samples occurred. Each scan takes 1.5±0.1 s including 0.5 s of 100 million recorded samples and 0.6 s of initial wait time. It can provide real-time data acquisition for slow-moving targets. An additional 0.5±0.3 s is incurred by calling MATLAB, which performs post-processing and visualizes the data in parallel with scanning, providing near-real-time visualization of range measurement.

Direct measurement of the peak power of the 5-ns RF pulse using a spectrum analyzer is challenging because of its short duration; a high-bandwidth, high-sampling-rate oscilloscope would provide more accurate temporal characterization. Therefore, the peak output power was estimated from a CW measurement at 2 GHz. According to Table 3, the measured output power for a TX amplitude of 0.3 was −19.2 dBm. Assuming linear amplitude scaling, increasing the TX amplitude to 1 increases the power by $20\log_{10}\left(1/0.3\right)=10.46$ dB, corresponding to an estimated output power of −8.74 dBm. With a TX gain setting of 30 dB, the corresponding theoretical level would be approximately 21.3 dBm; however, the X310’s maximum RF output power is approximately 10 dBm. Therefore, the actual peak output power is limited to approximately 10 dBm. No amplifier is used in this radar system (neither low-noise amplifier in receive path nor power amplifier in transmit path).

The radar scans the scene 20 times and updates a two-dimensional image. At each scan position, 100 consecutive impulses are transmitted sequentially over a short time interval and received by the RX channel. Fifty of the 100 received responses are selected and averaged to reduce random fluctuations in the received signal. Although the reflector is moving during these repeated acquisitions, they are collected over a sufficiently short time interval that the target displacement within a set of 50 acquisitions is small compared with the range sampling interval and the effective range resolution. Therefore, the target response remains concentrated within a small range interval during the averaging process, while random measurement fluctuations are reduced.

**Figure 9:**
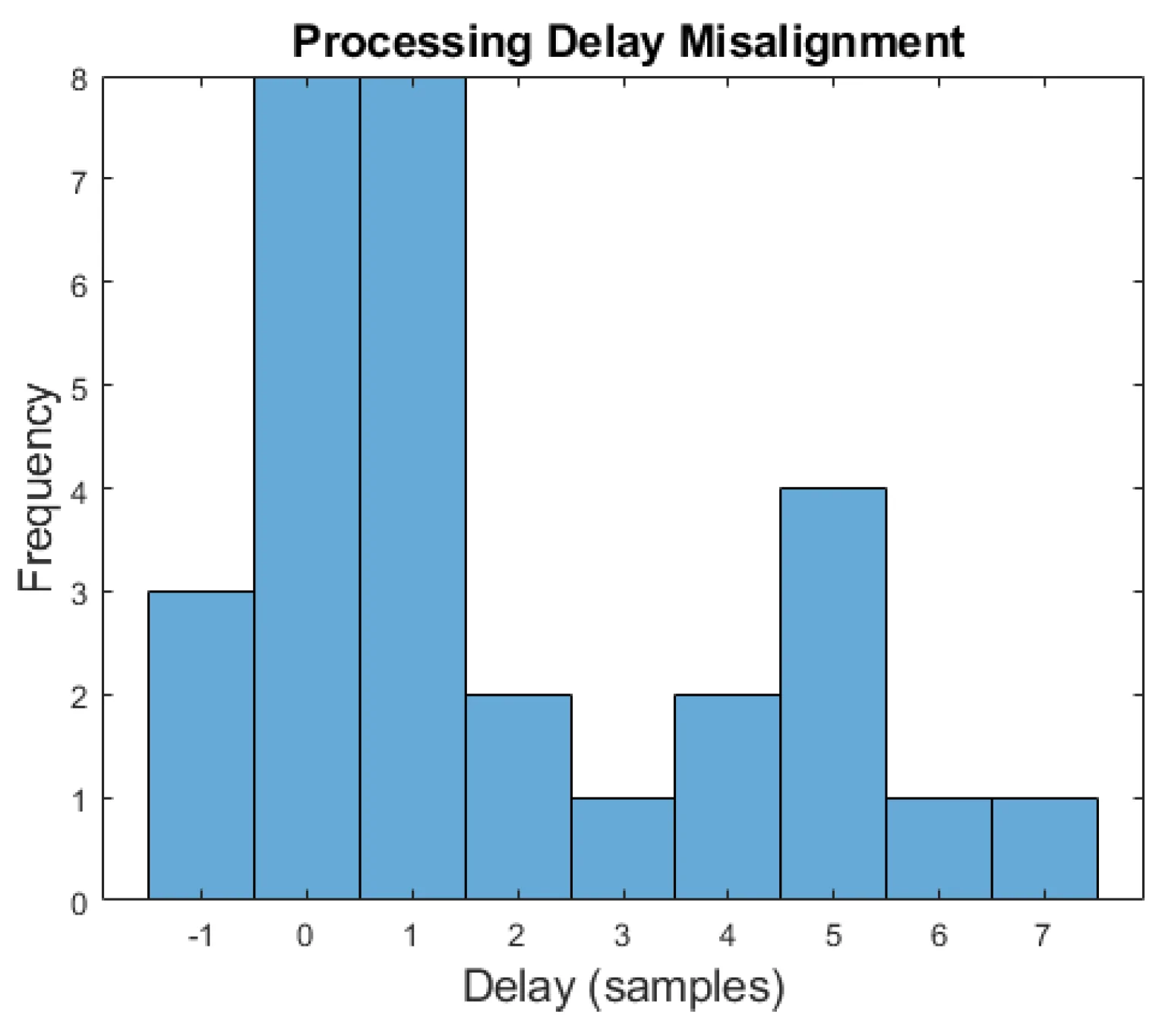
Histogram of processing delay misalignment for a total of 20 scans.

The resulting averaged signal provides a representative response for each scan position. These responses are then aligned to compensate for scan-to-scan timing variations. A small number of samples of misalignment can occur between consecutive scans because of timing jitter and variations in the data-acquisition and processing sequence. The measured inter-scan misalignment has a mean value of 8.8 ns and a standard deviation of 26.1 ns. The corresponding distribution of the measured timing misalignment is shown as a histogram in Figure 9.

The first scan, corresponding to a reflector placed at a known reference position, was selected as the temporal reference and calibration position. The magnitude of each subsequent response was cross-correlated with the magnitude of this reference response, and the lag corresponding to the maximum cross-correlation was used to estimate the relative timing offset between scans. The estimated offset was then compensated by applying the corresponding linear phase ramp in the frequency domain. The known reflector position at the first scan was used to establish the reference delay for subsequent range estimation, while the cross-correlation procedure was used to compensate for inter-scan timing variations.

The purpose of this alignment is to compensate for variations in the timing of the stationary system response rather than to remove or compensate for the physical propagation delay of the target echo. Because the transmitter and receiver remain fixed during the scan sequence, the dominant transmitter–receiver crosstalk occurs at approximately the same propagation delay for all scan positions. The cross-correlation therefore identifies the small relative shift of this stationary component caused by inter-scan timing variations. After the estimated shift is applied, the crosstalk is aligned to a common temporal reference across the scans. The physical delay of the reflector is not used as the alignment reference and is not forced to coincide with the reference position. Consequently, as the reflector moves, its echo remains at its corresponding propagation delay, and the change in its delay is preserved for subsequent range estimation.

After temporal alignment, the background response is estimated by averaging the data across multiple scans and subsequently subtracted to suppress stationary system components, particularly the strong transmitter–receiver crosstalk. Because these components occur at approximately the same delay after alignment, their contribution can be effectively reduced through background subtraction. In contrast, the target response changes in delay as the reflector moves through the measurement region. Therefore, the target response is not fully represented by the stationary background and remains visible after subtraction, allowing its range trajectory to be extracted from the resulting range profiles.

**Figure 10:**
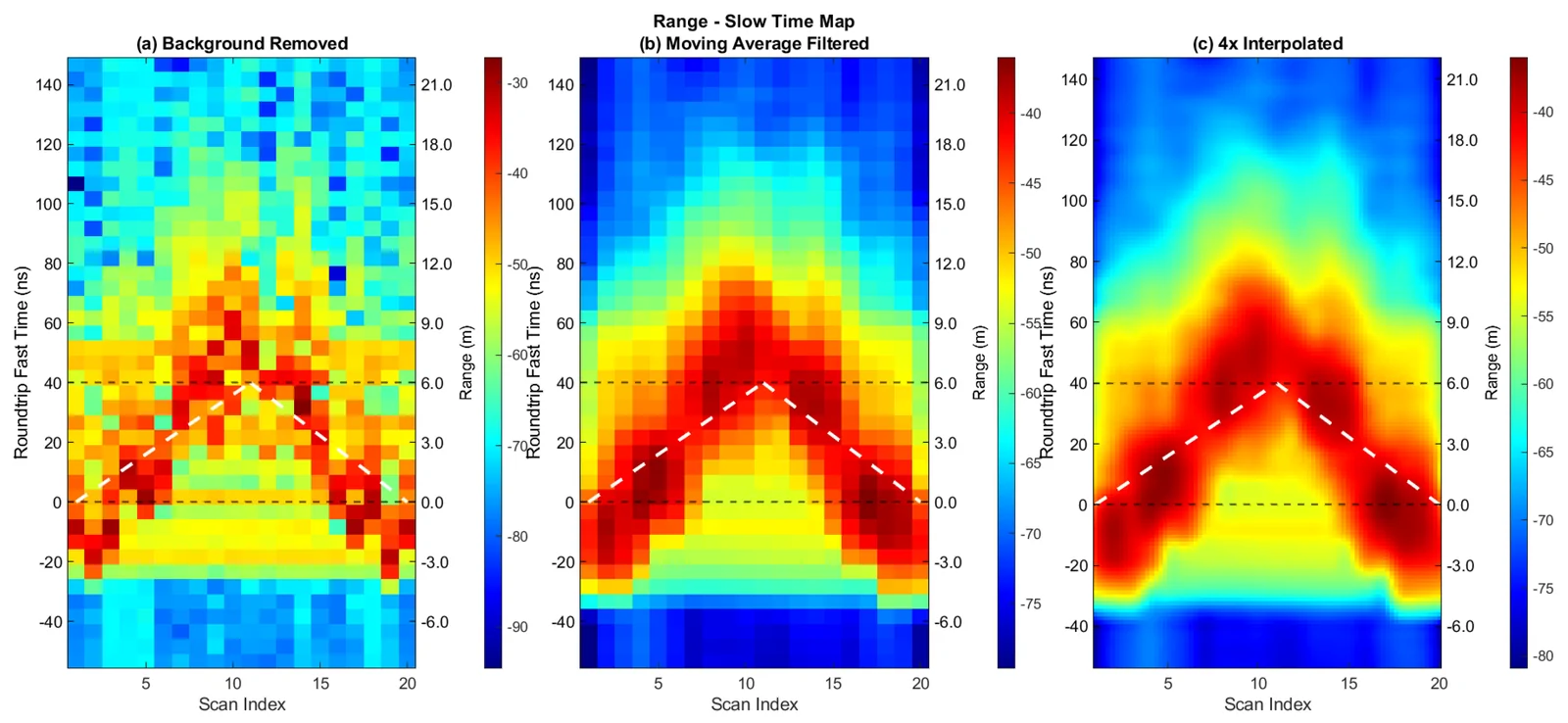
Range–slow-time plot for radar measurement: (**a**) background clutter is removed from raw data; (**b**) a 2D moving average is applied; (**c**) data is interpolated with a factor of 4 for both dimensions.

The background-removed data are plotted row by row, with each row representing one scan and the corresponding target response at that scan position. As the scan progresses, a new row is added to the range–slow-time plot, allowing the motion of the target to be visualized as a function of scan number. Figure 10 shows the resulting range–slow-time intensity plot, cropped to the region of interest. The white dashed line represents the ground-truth target trajectory. Figure 10a shows the amplitude of the background-removed data.

The horizontal axis represents slow time, expressed by the scan number. The left vertical axis represents fast time in nanoseconds and corresponds to the measured round-trip propagation time. The right vertical axis represents the corresponding one-way target range, calculated from the corrected propagation time using the speed of light in free space. The zero-delay reference corresponds to the location of target at first scan. The color bar represents the received-signal amplitude in dB.

For improved visualization and to reduce speckle noise and scan-to-scan fluctuations, the amplitude of the background-removed data was processed using a two-dimensional moving-average filter. The resulting smoothed data are shown in Figure 10b. Figure 10c shows a fourfold upsampled version of Figure 10b, obtained using linear interpolation in both the slow-time and range directions. These smoothing and interpolation operations are used to improve the visualization and facilitate extraction of the target trajectory; they do not increase the fundamental range resolution determined by the system bandwidth.

The target location at each scan was determined from the maximum-amplitude response within the region of interest. As shown in Figure 10c, the aluminum reflector moves approximately 6 m away from the antennas during scans 1–11 and then moves back toward the antennas during scans 12–20. The estimated target range was compared with the known ground-truth trajectory at each scan position. The ranging error was evaluated for each scan position, and the resulting RMSE, standard deviation, and maximum absolute error are summarized below.

For the background-removed data, the RMSE, standard deviation, and maximum absolute error are 1.99 m, 2.03 m, and 3.95 m, respectively. After applying the two-dimensional moving-average filter, these values decrease to 1.75 m, 1.79 m, and 2.45 m, respectively. For the fourfold-interpolated data, the corresponding values are 1.75 m, 1.76 m, and 2.65 m. The reduction in RMSE and maximum error after smoothing indicates that the moving-average filtering reduces scan-to-scan fluctuations and improves the consistency of the extracted target trajectory. The nearly unchanged RMSE after interpolation indicates that interpolation primarily provides a denser visual representation of the trajectory rather than improving the underlying ranging accuracy or fundamental range resolution. Although this error is larger than the theoretical range resolution and roughly 29% of the total target displacement, the result reflects the practical ranging performance of the complete measurement system under the experimental conditions.

The experiment demonstrates that the implemented software-defined radar can detect and track the motion of the reflector over the investigated range. The quantitative comparison with the known target trajectory provides an experimental measure of the ranging error, while the observed RMSE values characterize the practical ranging performance under the measurement conditions.

The software-defined radar based on the USRP X310 and UBX-160 was successfully implemented using the available sampling rate and RF bandwidth. The measured target delays and resulting range trajectory are consistent with the expected propagation behavior and demonstrate the capability of the system for range measurement and target’s motion visualization.

## 6. Conclusions

This paper presented a high-resolution impulse radar implemented on a USRP X310 software-defined radio equipped with a UBX-160 daughterboard. By using the low-level UHD C++ API and carefully configuring the host computer and network interface, real-time operation for slow-moving targets at 200 MS/s and wide instantaneous bandwidth were achieved. A single-channel architecture with timestamping was shown to provide accurate ranging without requiring a second RF channel.

The proposed system provides a flexible, reproducible platform, detailed in its requirements and limitations, for wideband SDR-based radar research with high resolution. The round-trip range resolution is 2 m with an accuracy of ±1.75 m. The visualization of target trajectory is updated within 2 s. Future work will investigate more advanced time-domain radar architectures and compare their performance with frequency-domain approaches.

## Figures and Tables

**Table 2:** Different configurations of host PC used in this study.

	Desktop	Laptop	Mini PC
CPU	12th-generation Intel Core i7-12700KF	Intel Core Ultra 7 155H	Core i9-13900H
RAM	64 GB	16 GB	64 GB
SSD	Samsung 870 EVO 2TB	1 TB	Samsung 990 EVO Plus 4 TB
Network	Intel Ethernet Controller X550-T	Two Sonnet Solo 10G Thunderbolt3-to-10-GbE adapters	Upgraded to Intel Ethernet Controller X550-T
Relative Overall Performance	Great	Medium	Great
Other	Bulky	Small, needs network adapters	Small

**Table 3:** Output power of USRP X310 at each center frequency.

Frequency (GHz)	Power (dBm)
0.5	−14.75
0.75	−15.7
0.8	−16.1
0.9	−16.5
1	−17.1
1.2	−17.6
1.5	−18
2	−19.2
2.5	−20.9
3	−22

## Data Availability

The data presented in this study are openly available in [GitHub] [https://github.com/skfabd/Impulse_Radar_on_USRP.git], accessed on 24 August 2026 [25].
